# Sleep and Exercise in Emergency Medicine Residents: An Observational Pilot Study Exploring the Utility of Wearable Activity Monitors for Monitoring Wellness

**DOI:** 10.7759/cureus.2973

**Published:** 2018-07-12

**Authors:** Zafrina Poonja, Preston O'Brien, Elfriede Cross, Rhonda Bryce, Erica Dance, Priya Jaggi, Joel Krentz, Brent Thoma

**Affiliations:** 1 Emergency Medicine, University of Alberta, Edmonton, CAN; 2 College of Medicine, University of Saskatchewan, Saskatoon, CAN; 3 Clinical Research Support Unit, University of Saskatchewan, Saskatoon, CAN; 4 Physical Education, Brandon University, Brandon, CAN; 5 Emergency Medicine, College of Medicine/University of Saskatchewan, Saskatoon, CAN

**Keywords:** physical activity, sleep, sleep, wearable activity monitor, emergency medicine, resident wellness, fitbit, physician wellness

## Abstract

Introduction

Burnout is well-documented in residents and emergency physicians. Wellness initiatives are becoming increasingly prevalent, but there is a lack of data supporting their efficacy. In some populations, a relationship between sleep, exercise, and wellness has been documented; however, this relationship has not been established in emergency medicine (EM) residents or physicians. We aim to determine whether a wearable activity monitor is a feasible method of evaluating exercise and sleep quality and quantity in emergency medicine residents and if these assessments are associated with greater perceived wellness.

Methods

Twenty EM residents from two training sites wore a wearable activity monitor (Fitbit ChargeTM, Fitbit, Inc., San Francisco, CA, USA) during a four-week EM rotation. The Fitbit recorded data on sleep quantity (minutes sleeping) and quality (sleep disruptions), as well as exercise quantity and quality (daily step count, daily active minutes performing activity of 3 - 6, and > 6 metabolic equivalents). Participants completed an end-of-rotation Perceived Wellness Survey (PWS), which provided information on six domains of personal wellness (psychological, emotional, social, physical, spiritual, and intellectual). PWS levels were compared between groups of subjects with higher or lower levels of activity and sleep (i.e., above and below the median subject-averaged values) using the Mann-Whitney U test. Other subject characteristics were similarly assessed for their association with PWS. When a possible confounding effect was seen, the data was stratified and reviewed using a scatterplot.

Results

Of the 28 eligible residents, 23 agreed to participate. Of these, 20 and 16 wore the device for at least 50% of the respective days and nights during the observation period. Two devices were lost. One PWS was not completed. There was no statistically significant correlation between resident perceived wellness survey scores, sleep interruptions, average daily sleep minutes, daily step count, or average daily active minutes for the sample overall. However, first-year residents and residents from years two to five reported different median PWS scores of 13.9 and 17.1, respectively. Further exploration by the training group suggested that step counts may correlate with wellness in participants in their first year of residency, while the quantity of sleep may have an association with wellness in participants in years two through five of their residency.

Conclusion

Using wearable activity monitor devices to capture sleep and exercise data among residents does not seem to be an effective approach. Our data does not support our hypothesis that overall resident wellness was associated with exercise and sleep quality and quantity as measured by such a device. These results are counterintuitive and may be complicated by several measurement-related limitations and the possibility that benefits depend on the stage of training.

## Introduction

Researchers to date have failed to agree on a single unifying definition of wellness [1]. However, it is broadly recognized that wellness is more than just the absence of disease [1]. The World Health Organization recognizes three key aspects of wellness: physical, mental, and social [2], while others have added additional dimensions, such as intellectual and spiritual wellness, to their definition [3-4].

Among physicians, workload expectations, such as long hours and shift work, can add extra challenges to maintaining wellness. Furthermore, the training expectations and the unpredictable nature of residency can lead to residents prioritizing work and learning over personal health and wellness. This stress can lead to depression, fatigue, and burnout [5]. Burnout and emotional exhaustion are common in emergency medicine [6-7]. In addition, shift work has been shown to have negative health implications [8]. Both sleep and exercise have been found to relate to wellness in other populations [9-10] and are particularly challenging to maintain on a shift work schedule [11]. These problems also affect residents [12].

Wellness initiatives addressing these various dimensions are becoming increasingly prevalent [13]. It is hoped that they will reduce the stress, depression, and burn-out frequently documented in medical residents and emergency physicians. However, their effectiveness has rarely been evaluated.

Commercially available wearable activity monitors, such as the Fitbit™ (Fitbit, Inc., San Francisco, CA, USA), are marketed as tools that can assess physical wellness. These devices measure surrogate markers for physical wellness, such as total sleep time, sleep interruptions, and exercise markers, such as step counts, speed, and heart rate [3-4, 14]. One study has attempted to assess the influence of Fitbit devices on exercise activity of emergency medicine (EM) residents, but it did not assess the impact of exercise on the overall wellness of its participants [15].

We evaluated the feasibility of using a wearable activity monitor to capture sleep and activity levels among residents and to determine whether these metrics are associated with the self-perceived wellness scores of EM residents during a four-week EM rotation. We hypothesized that exercise and sleep quantity and quality will correlate positively with an overall self-assessment of resident wellness as measured by a validated wellness survey. If this correlation can be demonstrated, wearable activity monitors could serve as a tool to assist in the evaluation of resident wellness initiatives.

## Materials and methods

Ethical approval was received from the University of Saskatchewan (UofS) (Beh 16-132) and University of Alberta (UofA) (Pro00063883) Research Ethics Boards.

Participants

All residents of the UofS and UofA Fellows of the Royal College of Physicians and Surgeons of Canada (FRCPC) and Canadian College of Family Physicians EM (CCFP-EM) programs were invited to participate in the study via email (50 residents between both sites). After obtaining informed consent, participants were provided with a Fitbit Charge (Fitbit Inc., San Francisco, CA) and asked to wear the device 24 hours per day, seven days per week throughout a four-week emergency medicine rotation during the period of July 1, 2016 to December 31, 2016. Each device was assigned a generic name and password to anonymize user data. The device was linked to the participant’s personal cell phone prior to the beginning of the study period.

Data collection

A study author (PO) exported device data from the Fitbit online portal to a spreadsheet. Fitbit usage was monitored three times per week to ensure that the data had been successfully logged. If missing data was noted, participants were contacted via email to address potential device usage issues (e.g., compliance with wearing device). Participants also completed a brief weekly survey detailing their daily Fitbit use. This provided an opportunity to assess device compliance, monitor technical difficulties, and provide context for missing data.

Exported data included metrics for sleep and exercise. Sleep quantity and quality were assessed by the average number of minutes of sleep per 24-hour period and the average number of awakenings per hours slept in a 24-hour period. Sleep periods were identified by the device based on decreased movement in a one hour period. Physical activity quantity and quality were assessed by the average daily number of steps and the average number of minutes of moderate to vigorous physical activity (> 3 metabolic equivalents) in a 24-hour period. These moderate and vigorous active minutes were summed as daily active minutes.

After the four-week EM rotation, residents were asked to complete the Perceived Wellness Survey (PWS), a wellness assessment tool consisting of 36 questionnaire items with documented validity evidence in other contexts [16]. The survey contains questions addressing six domains: psychological, emotional, social, physical, spiritual, and intellectual wellness. Each question was scored from 1 - “very strongly disagree” to 6 - “very strongly agree.” A score for each domain was provided and combined with other domains to provide a measure of overall wellness as per PWS scoring instructions. Demographic information for each participant was collected alongside the PWS survey and included age, gender, program location, training program (either the Fellow of the College of Physicians and Surgeons of Canada (FRCPC) or the Certification in the College of Family Physicians - Emergency Medicine (CCFP-EM) program), postgraduate year (PGY), marital status, and number of dependents.

Data analysis

Descriptive statistics were calculated to describe the sample, utilizing frequencies and proportions for categorical values and medians with interquartile ranges for continuous variables. Subjects’ values for a specific activity/sleep parameter were considered missing if data for the parameter were not recorded for at least 50% of the dates during the 28-day period. Participants with insufficient device data and those who did not complete the PWS were excluded from the related analyses. The metrics for the included participants were averaged across the number of dates during which the device was worn to provide average measures for each participant.

The averaged sleep and physical activity measurements of all included participants were then stratified at the median into low and high levels; PWS scores were compared between these low and high groups using the Mann-Whitney U test. To identify potential confounding influences, PWS was similarly compared between levels of categorical covariates and between low and high groupings for continuous covariates, the latter again stratified at the median value. Although limited by the small sample size, if the PWS was found to differ by levels of a covariate (i.e., potential confounder), the data were stratified by the covariate groups to remove the confounding influence of the factor, and the existence of a relationship between the PWS and Fitbit metric was reevaluated via scatterplot and Spearman correlation. 

## Results

Among the 20 participants with adequate data for assessment, eight were from the UofS and 12 from the UofA. The median age was 28 years. The majority were female (60%), single (60%), and had no dependents (90%) (Table 1). Fifty-five percent were in their first postgraduate year of training and 85% were in the FRCPC residency program.

**Table 1:** Subject Characteristics ​​​​​​​IQR: interquartile range; FRCPC: Fellow of the Royal College of Physicians of Canada; CCFP-EM:  Certificate of the College of Family Physician (Emergency Medicine); PGY: postgraduate year; PWS: Perceived Wellness Survey *Includes three subjects in common-law relationship; †Only available in 16 of 20 subjects (subjects missing more than 50% of sleep records were not included) ‡Calculated for each individual as the number wakings recorded within a given day/number of sleep hours within the day, averaged across all sleep records for the individual

Categorical variables, n (%)			
Sex	Male	8 (40.0)	
	Female	12 (60.0)	
Marital status	Partnered*	8 (40.0)	
	Single	12 (60.0)	
Children	Yes	2 (10.0)	
	No	18 (90.0)	
Program	FRCPC	17 (85.0)	
	CCFP-EM	3 (15.0)	
Program Year	PGY 1	11 (55.0)	
	PGY 2	3 (15.0)	
	PGY 3	4 (20.0)	
	PGY 4	0 (0)	
	PGY 5	2 (10.0)	
Location	University of Saskatchewan	8 (40.0)	
	University of Alberta	12 (60.0)	
Continuous variables, median (IQR)			
Age, years		28 (27, 30)	
Average daily step count		8,566 (6,745, 10,543)	
Average daily activity minutes		226 (164, 264)	
Number of activity records per subject		26 (24, 26.5)	
Average daily time in bed, minutes		448.6 (401.9, 464.2)	
Average daily sleep minutes†		417 (379, 435)	
Average daily number of awakenings per hours sleep†‡		1.0 (0.2, 2.5)	
Number of sleep records per subject†		21.5 (18.0, 24.5)	
PWS score		15.1 (13.4, 17.2)	

**Figure 1:**
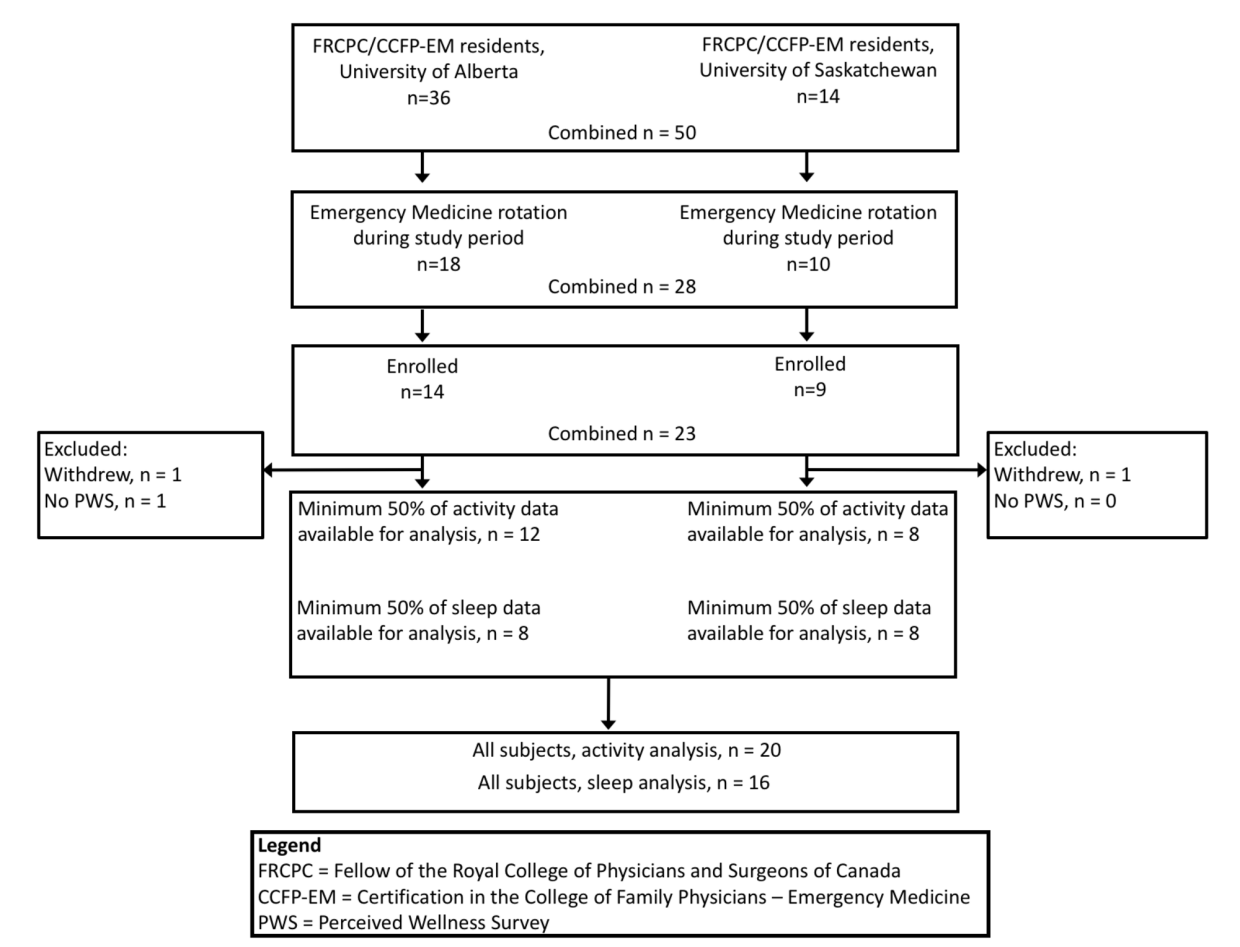
Participant flow diagram n: number

Participants were recruited and analyzed as outlined in Figure 1. Three participants were excluded from data analysis: two lost their Fitbit devices and elected to discontinue their study participation and one did not complete the PWS.

For all participants, the median averaged daily active time was 226 minutes (3.8 hours) and daily step count was 8,566 (approximately 6 - 7 km/day). The median averaged daily sleep minutes was 417 (7.0 hours) and the median averaged daily number of awakenings was 1.0 per hour of sleep. The median overall PWS score was 15.1 of a maximum 28.8.

Of those subjects with an average daily step count ≥ the 50th percentile, the median PWS was 15.8 compared to 15.0 for those who took fewer steps on average (p = 0.58). Similarly, for those whose average daily activity minutes were at the 50th percentile or higher, the median PWS was 14.6 compared to 13.9 for those below the 50th percentile (p = 0.97).

The median PWS among subjects whose average daily sleep minutes were greater than or equal to the 50th percentile was 15.6, compared to 14.2 for those who typically slept less (p = 0.65). Among those who typically woke on average at least once per hour slept, the PWS was 14.3, compared to 16.3 for those whose average nightly awakenings were less than the median value (p = 0.44). 

There was no significant association between PWS scores, sleep interruptions, averaged daily sleep minutes, daily step count, or average daily active minutes for the sample overall (Table 2).

**Table 2:** Median PWS Values by Predictor Status

Table 2. Median PWS values by predictor status				
			Outcome	
		n (%)	PWS, median (IQR)	p-value*
Overall		20	15.1 (13.4, 17.2)	-
By key predictors				
Average daily step count	≥ 50th percentile†	10 (50.0)	15.8 (13.9, 17.2)	0.58
	< 50th percentile	10 (50.0)	15.0 (12.9, 17.1)	
Average daily active minutes	≥ 50th percentile‡	10 (50.0)	14.6 (13.0, 17.0)	0.97
	< 50th percentile	10 (50.0)	13.9 (11.9, 16.3)	
Average daily time in bed	≥ 50th percentile‡	8 (50.0)	15.6 (14.2, 17.7)	0.38
	< 50th percentile	8 (50.0)	14.7 (13.0, 16.9)	
Average daily sleep minutes	≥ 50th percentile§	8 (50.0)	15.6 (14.2, 17.0)	0.65
	< 50th percentile	8 (50.0)	14.2 (13.0, 17.1)	
Average daily number of awakenings per hour sleep**	≥ 50th percentile|	8 (50.0)	14.3 (13.0, 16.3)	0.44
	< 50th percentile	8 (50.0)	16.3 (14.1, 17.1)	
By additional covariates				
Age, years	≥ 50th percentile††	10 (50.0)	14.7 (13.4, 17.1)	0.94
	< 50th percentile	10 (50.0)	15.8 (13.9, 17.1)	
Sex	Male	8 (40.0)	14.4 (10.9, 14.4)	0.31
	Female	12 (60.0)	15.1 (13.9, 17.3)	
Marital status	Partnered‡‡	8 (40.0)	16.2 (13.4, 17.3)	0.52
	Single	12 (60.0)	14.6 (12.4, 17.0)	
Children	Yes	2 (10.0)	15.1 (12.9, 17.2)	0.95
	No	18 (90.0)	15.1 (13.8, 17.1)	
Program	FRCPC	17 (85.0)	14.7 (13.8, 17.2)	0.77
	CCFP-EM	3 (15.0)	15.4 (14.2, 16.2)	
Year in program	PGY 1	11 (55.0)	13.9 (11.9, 16.2)	0.07
	PGY 2-5	9 (45.0)	17.1 (14.7, 17.4)	
Location	University of Saskatchewan	8 (40.0)	15.6 (13.4, 17.2)	0.91
	University of Alberta	12 (60.0)	14.6 (13.4, 17.2)	
PWS: Perceived Wellness Survey; IQR: interquartile range; *Mann-Whitney U test; †50th percentile = 8,566 steps; ‡50th percentile = 226 minutes; §50th percentile = 417 minutes, not available in four subjects; |50th percentile = 1.0 awakenings per sleep hour, not available in four subjects; **Calculated for each individual as the number of wakings recorded within a given day/number of sleep hours within the day, averaged across all sleep records for the individual ††50th percentile = 28 years; ‡‡Includes three subjects in common-law relationship				

**Figure 2:**
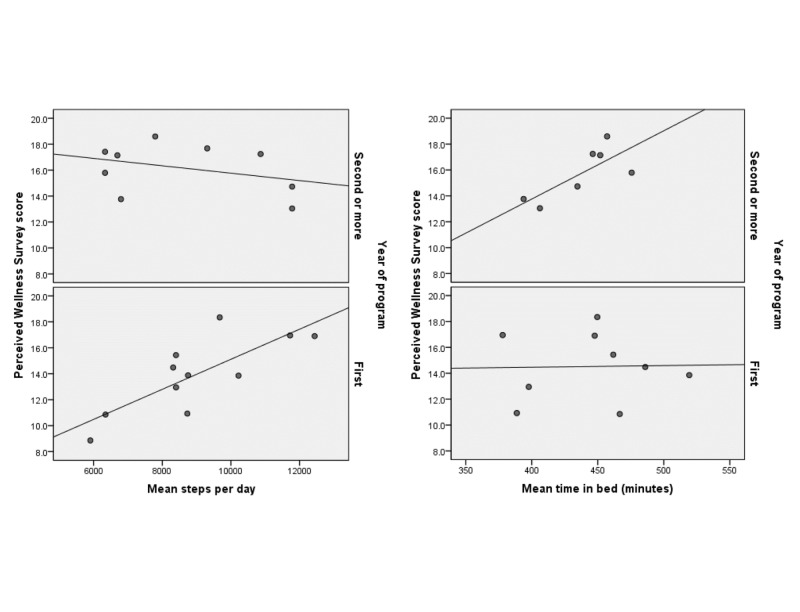
Respective scatter plot of average step count and time in bed, by postgraduate year of training

When evaluating PWS scores by covariate status, there was a trend toward higher median wellness scores in PGY2-5 residents versus PGY1 residents (17.1 versus 13.9, p = 0.07). When evaluated separately by PGY grouping, a moderate positive Spearman correlation was observed between the number of steps and the PWS score among PGY1 residents (correlation coefficient = 0.62), but not among PGY2 to 5 (correlation coefficient = 0.12). However, when the relationship between minutes in bed and PWS scores were evaluated within the separate PGY groups, a correlation between rest (time in bed) and perceived wellness correlates was suggested in PGY2 to 5 residents (correlation coefficient = 0. 57), but not in PGY 1 residents (correlation coefficient < 0.01). Relationships between all other Fitbit metrics and wellness were weak (correlation coefficient ≤ 0.31) when evaluated within either of the PGY groups. The scatterplots of these associations are presented in Figure 2.

## Discussion

Our pilot study had multiple limitations related to sample size, generalizability, and the wearable activity monitors themselves. While we are still able to generate several hypotheses with our data, we hope that studies exploring related questions in the future will learn from the challenges faced by using wearable activity monitors to study residents.

First, related to sampling, residency cohorts in EM residency training programs are relatively small. This limited the sample size of the pilot study despite the inclusion of a second site. One potential solution might be to broaden the study population into residents of all specialties since most residencies share lifestyle and wellness challenges. The restriction of our sample to EM residents allowed us to focus on the impact of shift work but limited the generalizability of our findings to other resident groups and locations.

From a recruitment perspective, the voluntary nature of our sample may have recruited individuals with relatively good self-care and a positive sense of wellbeing, thus limiting our ability to detect differences in wellness brought about by poorer practices. Similarly, it is possible that EM residents as a cohort are highly physically active at baseline, and as such, small differences between residents might not contribute to increased PWS scores.

Although the Fitbit measures physical activity metrics, such as step counts, these are metrics that are not necessarily produced by dedicated exercise activities. For example, during a busy EM shift, a resident could have high exercise and step count metrics and yet experience high levels of stress in a busy department, thus potentially invalidating the benefits of this physical activity. It was not feasible with the data that we obtained to further qualify the nature of participant activity. Additionally, given the short study period, participants may have increased their activity due to the Hawthorne effect [17] but had inadequate time to receive corresponding benefits to their wellness. The PWS does not provide reference values to indicate what might be considered poor, average, or above-average levels of perceived wellness.

Several limitations relate to the use of the activity monitor itself. While we had initially planned to monitor compliance using the online portal, this proved to be difficult as the device was not automatically synchronized to the participant’s device - it only synchronized if the application was opened or the device was actively synchronized by the participant. As such, it was difficult to tell whether participants were not wearing the device or simply had not opened the application on their mobile device. In addition, some data was lost if the device ran out of a charge without previously having been synchronized to the online portal. We identified this issue after the first cycle of participants and attempted to resolve it by reminding participants to sync their device on a regular basis, but not all participants complied with this request. Additionally, two participants lost their device while wearing it, as the clips on the wristband occasionally would become undone during regular activity (e.g., putting on and removing gloves).

The accuracy of the wearable activity monitor’s assessments was also questionable. One participant noted that they had accumulated > 1,500 steps during a long drive for which they had placed their device on the dashboard. It is possible that the device may over-report activity by sensing vibration patterns and interpreting them as physical activity. Similar inaccuracies have been noted in other settings [18]. Moreover, device use was limited in some water-related activities, given that it is a non-waterproof device. Some residents reported engaging in physical activities that are not reliably quantified by the device, such as swimming, biking, and horseback riding.

Similarly, there are concerns regarding the assessment of sleep metrics. A previous investigation has doubted the validity of wristband devices for monitoring sleep [4]. Specific to the EM experience, shift work and subsequent variation in sleep times may lead to a reduced quality of sleep overall that is not accurately reflected by minutes spent asleep or the number of awakenings, resulting in no association between wellness and measured sleep quantity/quality. It is of interest, however, that increased time-in-bed appeared to reflect improved wellness in more senior residents. As this metric potentially captures other leisure activities similar to lying in bed (e.g., relaxing on the couch, television watching, etc.), it may, in part, reflect personal time, low levels of which have been associated with reduced resident wellness [19].

Overall, our study findings suggest that the use of wearable activity monitors among EM residents is hampered by issues of compliance and potential measurement inaccuracy. The data collected in our study lacked solid statistical evidence to support the associations between measured wellness metrics and perceived wellness scores seen in other populations [9-10], although this relationship may have been obscured by the confounding and effect modification of the training stage. Our finding of poorer well-being in residents in earlier training stages has been observed in other studies [20-21], and given that stressors have previously been found to differ by the level of training [20], it is not completely surprising that the associations between wellness and both physical activity and time in bed differed by year of training. However, given the limited sample size and other noted concerns, all findings can only be viewed as exploratory.

Primarily, this pilot study provides valuable information regarding unforeseen limitations of activity monitors that could be mitigated by focusing on participant compliance and developing more rigorous data collection methods. It also suggests that future studies should evaluate the possibility that factors influencing resident wellness may differ by the stage of training. 

## Conclusions

In conclusion, the use of wearable activity monitors to capture sleep and exercise data was not an efficient approach in our population of emergency medicine residents. Additionally, we did not demonstrate a clear link between the metrics used to measure physical wellness and self-reported wellness scores in emergency medicine residents. It is possible that such predictors are training stage-specific or that one or more of the other wellness domains play a larger contributing role to overall wellness in this cohort. In order to develop effective wellness initiatives, it may be worthwhile to explore the contributions of psychological, emotional, social, spiritual, and intellectual wellness to overall self-reported wellness in residents and physicians.
